# Differential Th Cell-Related Immune Responses in Young Physically Active Men after an Endurance Effort

**DOI:** 10.3390/jcm9061795

**Published:** 2020-06-09

**Authors:** Dorota Kostrzewa-Nowak, Robert Nowak

**Affiliations:** Centre for Human Structural and Functional Research, University of Szczecin, 17C Narutowicza St., 70-240 Szczecin, Poland; robert.nowak@usz.edu.pl

**Keywords:** cytokines, fatigue, flow cytometry, physical activity, progressive effort, T lymphocytes

## Abstract

The participation of T cell subsets in the modulation of immunity in athletes triggered by maximal effort was investigated. In total, 80 physically active young men (range 16–20 years) were divided into 5 age groups: 16, 17, 18, 19, and 20 years old. They performed efficiency tests on mechanical treadmills until exhaustion. White blood cell (WBC) and lymphocyte (LYM) counts were determined, and the type 1 (Th1), type 2 (Th2) helper T cells, T helper 17 (Th17), and T regulatory (Treg) cell distribution and plasma levels of selected cytokines were analyzed. An increase in WBC and LYM counts after the test and in Th1 and Treg cells after the test and in recovery was observed. There were no changes in Th2 cells. An increase in interleukins (IL): IL-2 and IL-8 was observed. The IL-6 level was altered in all studied groups. IL-17A and interferon gamma (IFN-γ) levels were increased in all studied groups. The mechanism of differential T cell subset activation may be related to athletes’ age. The novel findings of this study are the involvement of Th17 cells in post-effort immune responses and the participation of IL-6 in post-effort and the long-term biological effect of endurance effort.

## 1. Introduction

Physical activity (PA) is one of the significant factors that positively modulates the immune system. Therefore, PA represents an important component in preventing or delaying the occurrence of chronic disorders, including autoimmune disorders, immunodeficiency, cancer, and metabolic diseases, such as obesity, hypertension, osteoporosis, and osteoarthritis [1]. The benefits of PA on psychosocial and cognitive health have also been demonstrated [2,3,4]. In general, short-term efforts of moderate intensity have a positive effect on the modulation of the immune system, which is why they are proposed as components of therapy in patients with immunodeficiency, as well as in the elderly, obese patients suffering from cancer, and those with chronic viral infections, such as HIV [4,5,6,7]. Improvement of immunity due to regular exercise of moderate intensity can be attributed to a decrease in inflammation, maintenance of thymus mass at a sufficiently high level, changes in the distribution of mature and naïve immune cells in the blood, increased immune surveillance, and/or relief of mental stress [6,7].

In particular, the promotion of PA as a way to help maintain or improve health should minimize the risk of injury, which can occur after an excessive physical bout. Although the mechanisms leading to the modulation of the immune system are well described in the literature [8,9,10,11,12], it seems that sterile inflammation may be crucial when taking the post-workout immunomodulation into account. This phenomenon is known to be a response to a psychological and/or physical stressor capable of causing an innate immune response in the absence of stimulation by a pathogen [13,14].

It is well known that T cell subsets, including T helper (Th), T cytotoxic (Tc), and regulatory T cells (Treg), play an important role in immunity. These cellular components are responsible for coordinating and regulating immune responses and killing infection-causing pathogens [15,16]. T cells can be divided into Type 1 (T1, including Th1 and Tc1) and Type 2 (T2, including Th1 and Tc1) subsets based, in part, on the cytokines produced [17]. Interferon gamma (IFN-γ) and interleukin (IL)-2 and -12 are associated with T1 subsets and promote cell-mediated immunity, while anti-inflammatory cytokines, including IL-4, IL-5, and IL-13, which are essential for humoral immunity development, are associated with T2 subsets. The balance between T1 and T2 immunity seems to be one of the most important biological factors related to the risk of viral infection in physically active men, both athletes and non-athletes [17].

The CD4^+^FoxP3^+^ (Treg) cells are also involved in post-effort immune modulation because of their role as central anti-inflammatory regulators of the immune response [16,18]. The participation of T cells in the modulation of immunity during maximal effort has been described in athletes [19,20,21,22]. On the other hand, numerous physically active young men, even though they are not professional athletes, use professional training programs to improve their physical fitness.

The impact of different types of effort on the distribution of T1/T2 cell subsets is not a common subject of research. Our previous study indicated that there was a significant role of Th1 and Treg cell subsets in response to the progressive effort in junior soccer players. The changes in T1/T2 cell balance did not always occur in parallel with their counts and functions [17]. On the other hand, it is not clear whether or not the age and volume of the training units had a significant impact on the molecular pathways of the immune response in physically active young men. Modulation of immune responses and silencing of self-reactive T cells is carried out by Treg cells (CD4^+^FoxP3^+^). Treg cells are involved in rebuilding the T1/T2 balance because of their role in suppressing the activation of T cell effector function, mostly through the release of IL-10, a known anti-inflammatory cytokine [16,23]. It has also been shown that the imbalance between the secretion of proinflammatory and regulatory (anti-inflammatory and multifunctional) cytokines can cause a violent and aggressive inflammatory response resembling an in vivo immune response to primary antigens [15,17,24,25].

This study was designed to better understand the influence of progressive endurance effort on selected biological and immunological parameters as a comparison of Th cell-related immune responses. The main goal of the study was to evaluate the participants’ Th cell-related immunological response to the same type of physical effort. Additionally, the examination of whether these changes differ in regards to the participants’ age was planned. To address this problem, different age groups of physically active young men performed the endurance effort test on a mechanical treadmill, and the distribution of CD3^+^CD4^+^ (Th) cell subsets, namely Th1 (CD4^+^IFN-γ^+^), Th2 (CD4^+^IL-4^+^), Th17 (CD4^+^IL-17A^+^), and Treg (CD4^+^FoxP3^+^), were analyzed. Moreover, to determine the physiological response to this type of effort, the levels of proinflammatory (IL-2, IL-4, IL-6, IL-12p70), anti-inflammatory (IL-10), and multifunctional (IL-8, tumor necrosis factor alpha (TNF-α), interferon gamma (IFN-γ)) cytokines were analyzed.

## 2. Materials and Methods

### 2.1. Participants

In total, 80 men aged 16–20 years old, declaring at least 50 min of training per day, were recruited for this study. Additionally, the participants were divided into five age-dependent groups. All participants performed the efficiency test on a mechanical treadmill as described previously [20,21]. All participants were non-smokers and refrained from taking any medications or supplements known to affect metabolism. Moreover, they had no history of any metabolic syndrome or cardiovascular diseases. They also had no medically detected disorders of hormone levels or immune system failure.

The recruitment of participants consisted of informing them (and their parents, when appropriate) about the study, the exercise protocol, and inviting them to take part in it. All athletes not meeting the inclusion criteria (e.g., taking medications or supplements affecting metabolism, not giving us or later withdrawing the consent) were excluded from the study.

The study was approved by the Local Ethics Committee at the Regional Medical Chamber in Szczecin (no. 03/KB/VI/2017). Participants (and their parents, when appropriate) were fully informed of any risks and possible discomfort associated with the experimental procedures before giving their written consent to participate.

### 2.2. Progressive Test Protocol

All participants performed the progressive efficiency test until exhaustion on a mechanical treadmill that started with 5 min of warm-up running at a speed of 5 km/h. During the proper test, the speed increased by 2 km/h after each 3 min of the test until exhaustion, which is until each participant refused to run because of maximal fatigue. The advantage of the treadmill run over other tests until exhaustion is the possibility of using a stationary breath-by-breath gas exchange data analyzer to measure cardiorespiratory fitness variables as a function of increasing fatigue. The cardiorespiratory parameters (maximum oxygen uptake (VO_2_max), maximum heart rate (HRmax), maximum ventilation (VE), anaerobic threshold (AT), respiratory quotient (RQ), respiratory compensation (RC), maximal voluntary ventilation (MVV), metabolic equivalent (MET), and respiratory frequency (Rf)) were determined using a state-of-the-art breath-by-breath gas exchange data analyzer Quark CPET (Cosmed, Albano Laziale, Italy) [26].

The endurance tests in each of the studied groups were performed after two weeks of vacation time, when the participants were asked to refrain from physical effort, especially training units. All the tests were performed in Centre for Human Structural and Functional Research, University of Szczecin, Szczecin, Poland.

### 2.3. Methods

Body mass and body composition parameters (percentage of fat (FAT), fat free mass (FFM), and total body water (TBW)) of the participants were determined using a Body Composition Analyzer Tanita BC-418MA (Tanita, Tokyo, Japan).

Blood samples were obtained three times from the median cubital vein during the experiment: Before the test (pre-test), no longer than 5 min after the test (post-test), and about 17 h after the test, at the end of recovery time (recovery). At each time point, blood samples were collected in a 7.5-mL S-Monovette tube with ethylenediaminetetraacetic acid (EDTA K3, 1.6 mg EDTA/mL blood) (SARSTEDT AG & Co., Nümbrecht, Germany).

White blood cell (WBC) and lymphocyte (LYM) counts were determined immediately after the blood sampling and were analyzed using the hematology analyzer ABX Micros 60 (Horiba ABX, Warsaw, Poland).

To determine the Th cell subset distribution, lymphocytes were isolated from peripheral blood using density gradient separating medium (Corning, Manassas, VA, USA) and were frozen at −80 °C using the standard protocol for in vitro cell line storage [27,28,29] until further analysis.

**Figure 1:**
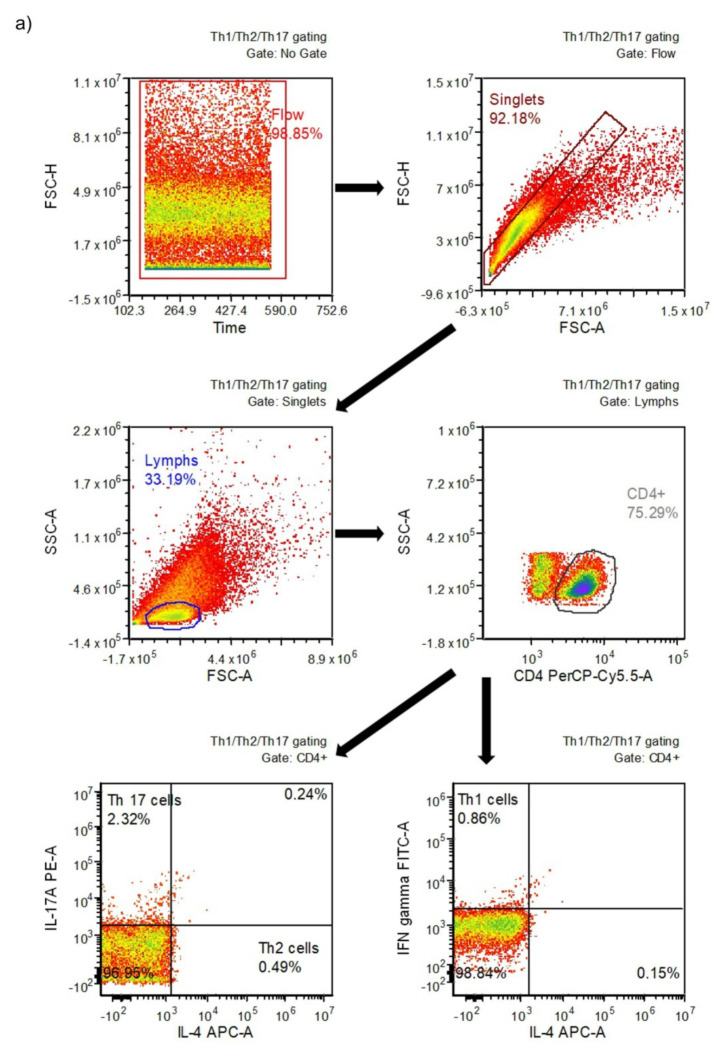

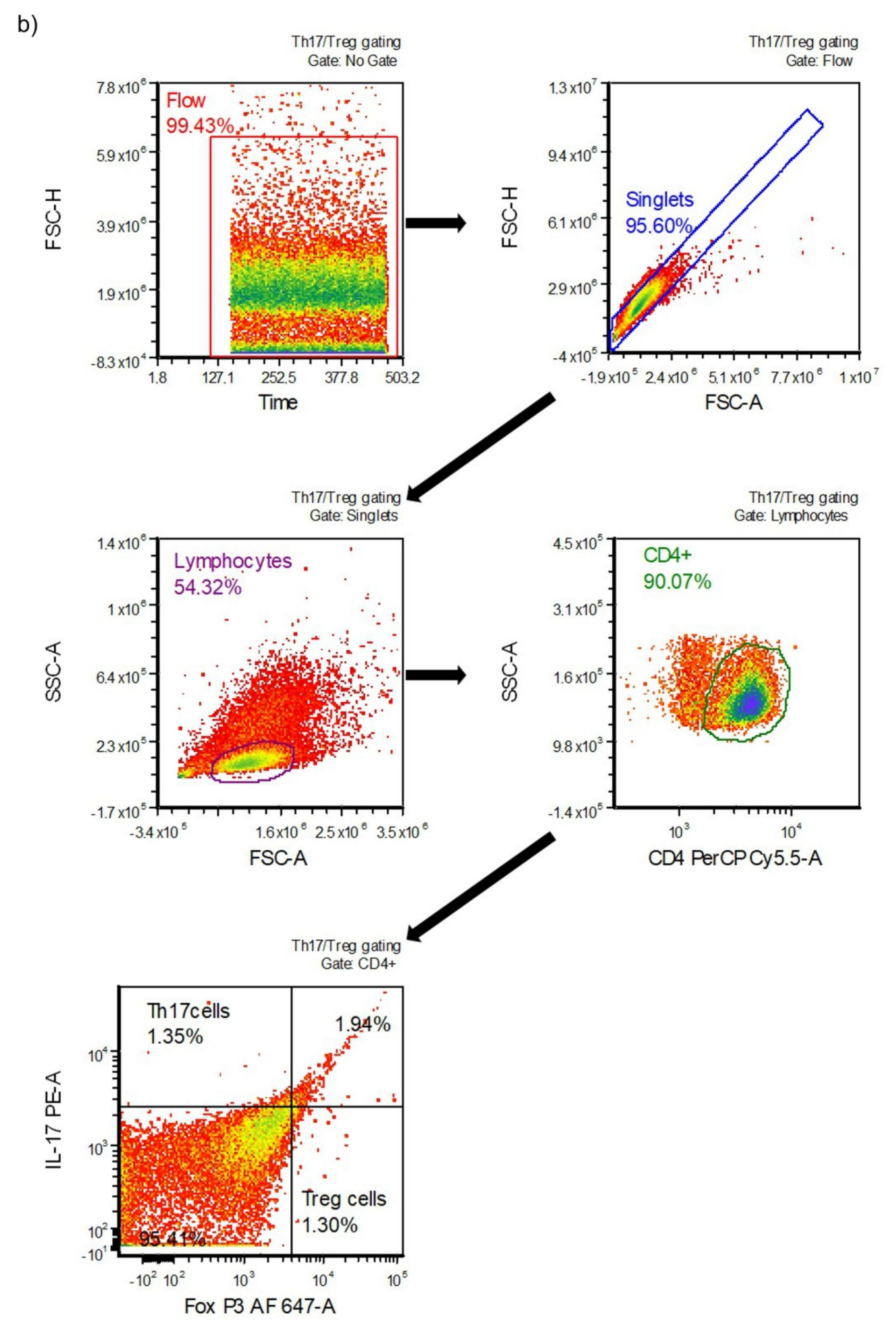
Gating strategy for determining different T cell subsets: (**a**) type 1 (Th1) and type 2 (Th2) helper cells; (**b**) T helper 17 (Th17) and T regulatory (Treg) cells.

To quantitate Th1 and Th2 cell subsets, the Human Th1/Th2/Th17 Phenotyping Kit (BD Biosciences, San Jose, CA, USA) and a BD Accuri™ C6 flow cytometer (Becton Dickinson, Franklin Lakes, NJ, USA) were used. Briefly, a cocktail containing FITC-labeled IFN-γ, PerCP-Cy5.5-labeled CD4, and APC-labeled IL-4 antibodies was used to determine the percentages of Th1 and Th2 lymphocyte subsets in isolated lymphocytes. The cells were fixed with BD Cytofix™ fixation buffer (BD Biosciences) (20 min, at room temperature), washed using BD Perm/Wash™ buffer (BD Biosciences), and resuspended in BD Perm/Wash™ buffer for 15 min (at room temperature in the dark). Thereafter, the buffer was removed, and the antibody-containing cocktail was added. After 30 min of incubation (at room temperature in the dark), the cells were washed with BD Perm/Wash™ buffer and suspended in stain buffer (FBS) prior to flow cytometric analysis. For each sample, the fluorescence signal of at least 10^4^ events gated for the forward and side light-scatter characteristics of lymphocytes was measured. The results were calculated using BD Accuri™ C6 (ver. 1.0.264.21) and FCS Express (ver. 4.07.0020 RUO Edition; De Novo Software, Los Angeles, CA, USA) software. The gating strategy for determining Th1 and Th2 cells is shown in Figure 1a.

To determine the Th17 and Treg cell subsets, the Human Th17/Treg Phenotyping Kit (BD Pharmingen™, San Jose, CA, USA) and the BD Accuri™ C6 flow cytometer were used. Briefly, an antibody cocktail to determine the percentages of Th17 and Treg subsets in isolated lymphocytes was used. The cocktail contained antibodies including PE-labeled IL-17A, PerCP-Cy5.5-labeled CD4, and Alexa Fluor^®^ 647-labeled FoxP3. The cells were fixed with Human FoxP3 buffer (20 min, at room temperature in the dark), than washed twice, and suspended in stain buffer (FBS). Thereafter, an appropriate aliquot of antibody cocktail was added, and the samples were incubated for 40 min (at room temperature in the dark). The cells were then washed twice with stain buffer (FBS) and analyzed. For each sample, the fluorescence signal of at least 10^4^ events gated on the forward and side light-scatter characteristics of lymphocytes was measured. The results were calculated using BD Accuri™ C6 and FCS Express software. The gating strategy for determining Th17 and Treg cells is shown in Figure 1b.

The measurement of selected cytokines, including IL-2, IL-4, IL-6, IL-8, IL-10, IL-12p70, IL-17, TNF-α, and IFN-γ, was performed using the BD Cytometric Bead Array (CBA) Human Inflammatory Cytokines Kit (BD Biosciences), BD™ Cytometric Bead Array (CBA) Human Th1/Th2/Th17 Cytokine Kit (BD Biosciences), and a BD Accuri™ C6 flow cytometer according to the manufacturer’s protocol. Briefly, an appropriate aliquot of mixed capture beads was prepared and incubated with serum enhancement buffer in the dark (30 min, at room temperature). Recombinant standards were diluted (in the rage of 0–1:1024) to prepare standard curves. Next, an appropriate aliquot of each dilution of recombinant standard or analyzed plasma sample was added to the capture beads, gently mixed and incubated 1.5 h in the dark (at room temperature). After washing the samples, the detection reagent was added, gently agitated, and incubated for another 1.5 h (in the dark, at room temperature). Finally, the samples were washed, fresh wash buffer was added, and the samples were analyzed by flow cytometry (BD Accuri™ C6). For each sample, the fluorescence signal of 2100 events gated for the capture bead population was measured. Results were calculated using FCAP Array™ Software (ver. 3.0.1; Soft Flow Hungary Ltd., Pecs, Hungary). The theoretical limit of detection for the cytokines provided by the assay manufacturer are presented in Table 1. The values below the threshold were obtained by extrapolating the values for sample intensities not falling within the limits of the standard curve. The inbound “force through zero” function during the creation of the standard curve was used.

### 2.4. Statistical Analysis

All data are presented as median (Q1-Q3). Statistical analysis was performed using STATISTICA (data analysis software system), version 13 software (2017; TIBCO Software Inc., Palo Alto, CA, USA; http://statistica.io). Significant differences between analyzed time points (pre-test vs. post-test vs. recovery) were calculated using Friedman’s analysis of variance for repeated measures followed by post hoc Dunn’s test with Bonferroni correction. Significant differences between age groups (16 vs. 17 vs. 18 vs. 19 vs. 20) were calculated using Kruskal–Wallis analysis of variance by ranks. The correlations between analyzed variables and the age of the participants were assessed using Spearman’s rank correlation coefficient determination. For each analysis, a *p*-value <0.05 was considered to be significant.

## 3. Results

Raw data obtained for each individual participant during the study are presented in Supplementary Material Table S1.

The participants were placed in the appropriate group based on their age. The baseline characteristics of all participants qualified for the study are presented in Table 2.

All studied groups were at a comparable sport level and had similar cardiorespiratory fitness measure values (Table 3). No significant differences were observed in the studied parameters: VO_2_max, HRmax, VE, AT, RQ, RC, MVV, MET, and Rf.

The analysis of WBC counts showed that progressive effort on the mechanical treadmill induced a significant increase in these parameters after the test in comparison to baseline and recovery values in all studied groups (Table 4). The post-test LYM counts were significantly higher in 17-, 19-, and 20-year-old participants (Table 4). There were no significant differences between the age groups at each time point. The results of lymphocyte immunophenotyping analyses showing the percentages of total lymphocytes, T, Th, Tc, NK and B cells are provided in the Supplementary Material Table S2.

**Figure 2:**
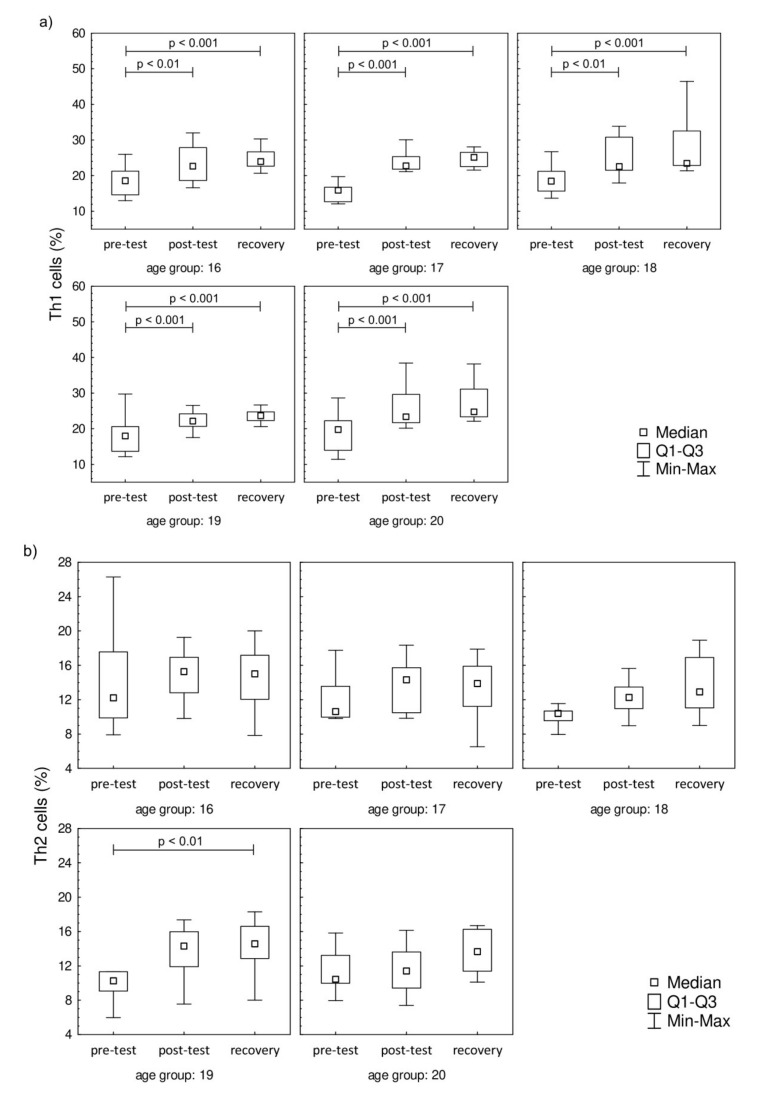

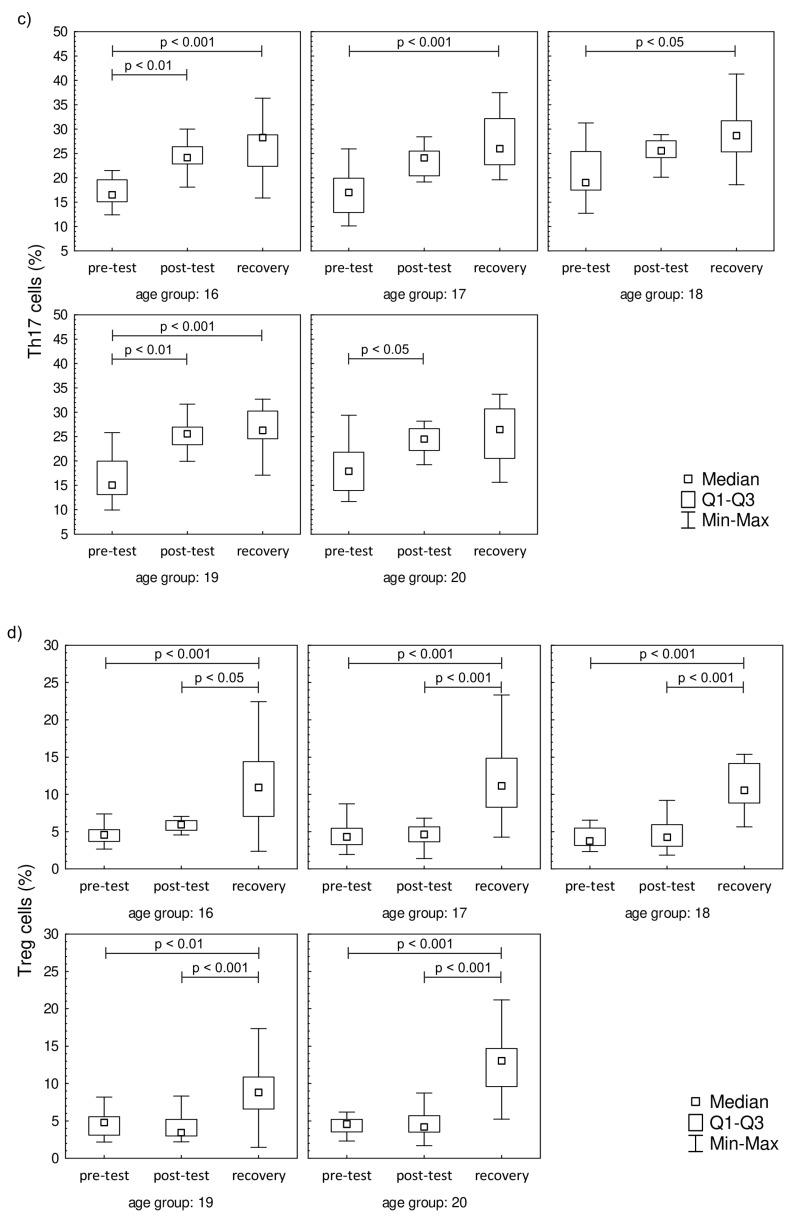
Th cell subsets in isolated lymphocytes from peripheral blood of the participants. (**a**) Th1 cell percentage; (**b**) Th2 cell percentage; (**c**) Th17 cell percentage; (**d**) Treg cell percentage. Significance levels of differences observed between analyzed time points (pre-test vs. post-test vs. recovery) were assessed using Friedman’s analysis of variance for repeated measures followed by post hoc Dunn’s test with Bonferroni correction.

Even though 17 h after the test the LYM counts were similar to pre-test values, there were differences in the percentage distribution of Th cells in the studied groups. The distribution of Th1 (CD4^+^IFN-γ^+^), Th2 (CD4^+^IL-4^+^), Th17 (CD4^+^IL-17A^+^), and Treg (CD4^+^FoxP3^+^) cells are presented in Figure 2. It was found that the endurance effort caused a significant increase in Th1 cell percentage in post-test and recovery time point in comparison to the pre-test value in all studied groups (Figure 2a). There were no baseline between-group differences in Th1 cell percentages. The progressive effort did not cause changes in Th2 cell percentage (Figure 2b). The post-test Th17 cell percentages were significantly higher in comparison to pre-test values in all age groups except for the 18-year-old participants (Figure 2c). The significant increase in this parameter was also observed during the recovery period in all studied groups with the exception of the oldest group (20 years old). The recovery values of Treg cells found 17 hours after the endurance effort were about 2-fold higher than baseline values in each studied group (Figure 2d).

**Figure 3:**
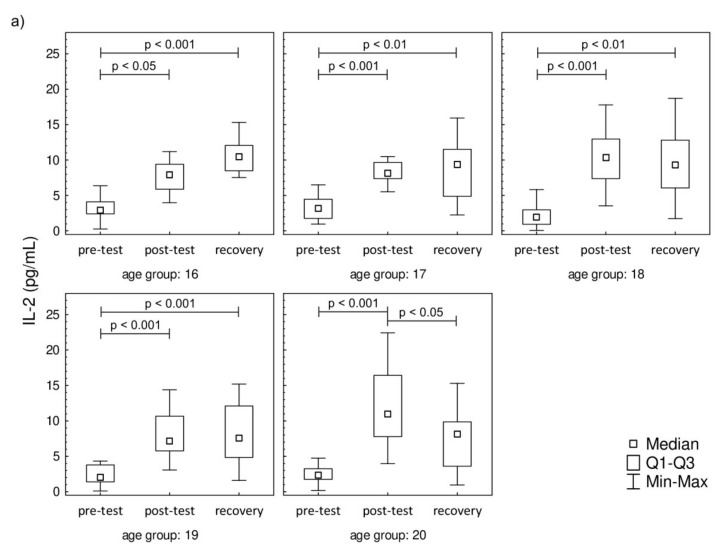

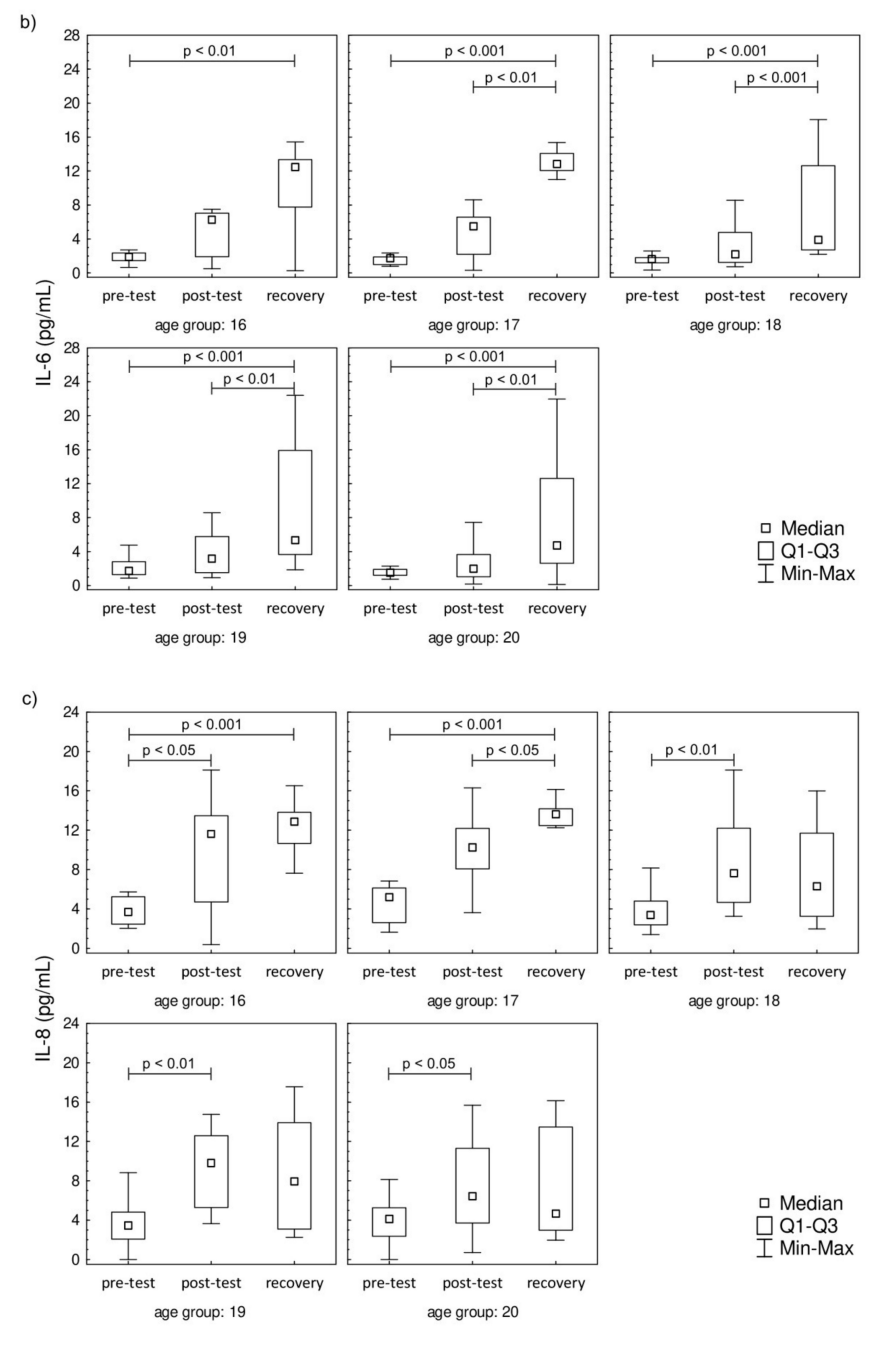

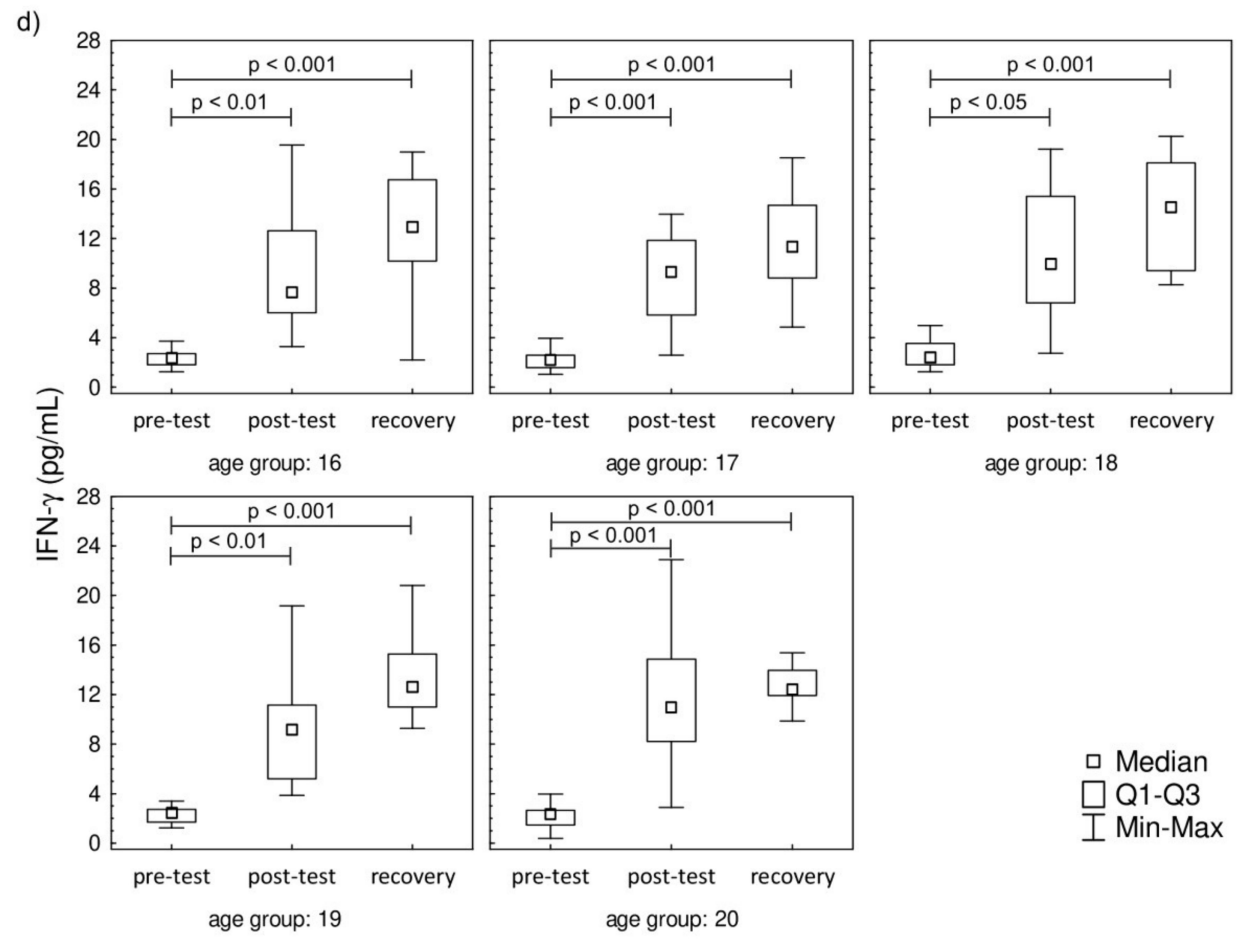
Median level of (**a**) interleukin-2 (IL-2), (**b**) interleukin-6 (IL-6), (**c**) interleukin-8 (IL-8), and (**d**) interferon gamma (IFN-γ) of participants’ plasma samples. Significance levels of differences observed between analyzed time points (pre-test vs. post-test vs. recovery) were assessed using Friedman’s analysis of variance for repeated measures followed by post hoc Dunn’s test with Bonferroni correction.

The plasma concentration values of IL-2, IL-6, IL-8, and IFN-γ are presented in Figure 3. The plasma concentration values of IL-4, IL-10, IL-12p70, IL-17A, and TNF-α that were below the threshold are presented in Supplementary Material Figure S1. A significant increase in IL-2 was observed post-test and in recovery, compared to baseline, in all studied groups. In contrast to IL-2, the IL-6 level was significantly higher in recovery in comparison to pre-test values in all studied groups. There were no changes in the post-test level of IL-6 in comparison to pre-test values. The increase in IL-8 was observed only during the recovery in comparison to pre-test, while the post-test values were not significantly higher in the 17-year-old participants. The increasing IL-8 trend in recovery was observed in 18-, 19-, and 20-year-old participants. It was also found that the progressive effort induced a significant increase in the secretion of IFN-γ among all studied groups both in post-test and recovery time points in comparison to pre-test levels.

The analysis of correlations between analyzed variables and the age of the participants revealed small to moderate correlation coefficients for different variables (Table 5). Only IL-12p70 and lymphocyte count correlated with participants’ age before the test. Significant correlations were observed in the case of Th2 and Treg cells’ percentages and IL-6, IL-10, IL-17A, and TNF-α levels at the post-test time point. The concentrations of IL-2, IL-6, IL-8, IL-10, IL-12p70, IL-17A, and TNF-α significantly correlated with the participants’ age at the recovery time point. However, the values of IL-4, IL-10, IL-12p70, IL-17A, and TNF-α are being at or below the threshold and should be treated with care. The linear regression analysis was also performed, and the results are presented in Supplementary Material Table S3. However, the low values of coefficients of determination must be emphasized, which at least in part may be explained by the narrow age range of the participants.

## 4. Discussion

Although there are numerous works regarding T cells, there is little data describing T cell subsets in regards of age groups of humans without athletic background (e.g., [30,31,32]). However, the age groups described are different and inhomogeneous. Louati et al. described the basic T cell distribution (T, Th, Tc, B, and natural killer (NK) cells) in the blood of donors divided into four age groups (19–25, 26–35, 36–45, and +45 years old, respectively) [31] and Holcar et al. analyzed lymphocyte subpopulations in three age groups, namely children (10 months to 11.4 years old), adolescents (15.5 to 20.9 years old), and adults (40.8 to 63.3 years old) [32]. Nevertheless, these studies did not investigate the changes in T cell subsets triggered by endurance effort.

### 4.1. The Role of Th1 and Th2 in Post-Effort Response

Leukocytosis and lymphocytosis are often described as the physiological effects of physical effort [33,34,35]. An increase in the leukocyte total count observed in this study was most probably caused by physical effort-related dehydration. Our previous study on athletes of a similar age preforming an endurance test on a mechanical treadmill showed an increase in post-test serum total protein that returned to the baseline value in the recovery time point [20]. An interesting observation found in our previous study was that there is an increase in Th cells after progressive effort in young physically active men [20]. Moreover, it was observed that this type of effort caused an increase in the Th1 cell subset [21]. The increase in naïve Th as well as Th1 cell percentages observed in our previous study [20,21] were the foundation of the present research, and the key role of Th1 cells in post-effort immune response was confirmed. The results of the present study showed that the participants’ age had no impact on Th1 cell recruitment as a biological effect of the physical effort as well as a long-term post-effort effect. Knowing the role of Th1 cells [16,17], it should be pointed out that the progressive effort did not induce a rapid inflammation state. This finding is the opposite of the literature data that indicate significantly increased levels of Th2-cell-related cytokines during recovery time, suggesting that post-effort changes during 3–72 h post effort were associated with Th2 cell response [36,37].

Taking the functional level into account, it was found that both Th1 and Th2 cell-related cytokines were secreted into the blood in the studied young men regardless of the age group, although not all of them were within the range of the assay. These observations are in line with the findings by Kakanis et al. [38]. Interestingly, they analyzed T cells stimulated with phytohemagglutinin and we observed the post-effort secretion of both Th1 and Th2 cell-related cytokines (IL-2, TNF-α, and IFN-γ or IL-4 and IL-6, respectively) without ex vivo cell stimulation. However, the Th1 and Th2 cell-related cytokine profiles were not similar in all studied groups. The Th1 cell distribution is in line with the secretion of Th1 cell-related cytokines in all studied groups. Since the IL-12p70 secretion is stimulated by TNF-α and IFN-γ [15,16,39], the recovery increase in the level of this interleukin confirms activation of Th1-related pathways in response to progressive effort. Moreover, the activation of this pathway is not related to the athlete age.

In the case of the Th2 cell-related cytokines, only the IL-6 level increased during the recovery in all studied groups and it negatively correlated with the age of the participants. In the oldest groups, an increase in Th2 cell-related cytokines was seen, yet there were no changes in Th2 cell percentage. Those findings are in line with our previous study, which showed no changes in Th2 cells’ distribution in junior groups of soccer players [21,22]. One of the probable explanations of this observation is that the changes in Th2 cell-related cytokines promote Th17 cell-related pathways of the post-effort immune response. On the other hand, these findings do not exclude the involvement of Th2 cells in the post-effort immune response. It is worth noting that the level of IL-6 is increased in all studied groups and this interleukin is responsible for co-activation and associated with the proliferation of T cells [40]. Moreover, IL-6 does not inhibit IL-2 production [40], which may explain the higher values of both interleukins found in our study even though a significant increase in Th2 cells was not observed. The fluctuations of the post-effort and recovery cytokine levels are related not only to the type of the effort [41,42] but also with the age of the participants (Table 5). The participation of IL-6, especially the long-term one (17 h after the completion of exercise), in the biological effect of an endurance effort is a novel finding of this study. The literature data demonstrate that regular exercise noticeably decreases TNF-α and IL-6 levels in the circulation, suggesting an anti-inflammatory effect of regular exercise [43,44]. The post-test correlation coefficients confirm the negative correlation between IL-6 and the age of the participants, which can also be extrapolated to longer training experience. Moreover, the same study suggests that endurance effort (on cycloergometers and the mechanical treadmill) does not stimulate the secretion of proinflammatory cytokines, including IL-6 [45,46,47]. On the other hand, an increase in plasma IL-6 seems to confirm the hypothesis that this interleukin released from contracting muscle during exercise acts in a hormone-like manner and mobilizes extracellular substrates and/or augments substrate delivery [39].

### 4.2. The Role of Th17 and Treg in the Post-Effort Response

The novel finding of this study is the involvement of Th17 cells in the post-effort immune response in physically active young men. It is well known that the presence of Th17 cells is associated with autoimmune diseases and long-term tissue inflammation [48,49,50,51]. According to the literature data, the promotion of Th17 cell differentiation is related to IL-6 increasing, which is in line with findings by other scientists [10,52]. The recovery time point increase in IL-6 in participants regardless of the age group may also explain the increase in the Th17 cell percentage.

One of Th17 cell differentiation factors is TGF-β. The same factor is responsible for differentiation of naïve T lymphocytes into regulatory T cells (Treg; CD4^+^FoxP3^+^) [49]. It is known that Treg cells are responsible for immunologic self-tolerance by inhibiting autoimmune responses and suppressing activated effector T and antigen-presenting cells, and this is mediated by numerous mechanisms [53,54]. From this point of view, the changes in Treg cell distribution are possibly related to the homeostatic role they play, and evidence suggests that inflammation, being a consequence of progressive effort, is a short-term phenomenon not related to the age of physically active men. The recovery increase in the Treg cell percentage is evidence that they play a key role as anti-inflammatory regulators of the long-term biological effect of progressive effort, at least in the case of examined young men. Clifford et al. found that the post-marathon values of peripheral Treg cells were lower than baseline levels, and also saw a rise over the baseline a day after the strenuous and high-intensive effort [23]. On the other hand, there are studies demonstrating a significant decrease in Treg cells after the half ironman triathlon and marathon [23,54]. Those findings are not in line with our observation. The most probable explanation of this inconsistency is related to the intensity and duration of the studied effort. The novel finding of our study is the observation that the change in Treg cells is not related to the participants’ age at least in the narrow range of 16–20 years. In summary, our study demonstrated that Th1 cells are related to a fast immune response, while in the long-term response, the Treg cells play a key role, and are likely differentiating under the influence of Th17 cells.

### 4.3. The Role of IL-8 in Post-Effort Response

The molecular mechanism of IL-8 is related to the transmembrane receptor for the chemokine CXC activated by the mitogen-activated protein kinase cascade. This cytokine is involved in angiogenesis, survival, and proliferation pathways [55,56]. Our previous study showed that an increase in IL-8 observed after progressive effort is one of the key anabolic effects of this type of effort in young soccer players [20,21]. Nieman et al. reported that high-intensity cycling caused a significant increase in IL-8 mRNA in peripheral leukocytes up to 1 h after the test [57]. Those findings help to explain the results found for the youngest (16 and 17 years old) participants in our study.

The involvement of IL-8 in hematopoiesis [55,56] during the recovery time observed in the youngest (16 and 17 years old) groups is in line with our previous study and strengthens the hypothesis that it may be the functional explanation for the release of CD4^+^ naïve T cells into circulation observed in other experiments [20,21]. This mechanism seems to be related to the age and training experience of the athletes, especially that there was a negative correlation (R = −0.32; *p* = 0.003) between the IL-8 plasma level and the age of the participants in the recovery time point. The lack of a significant increase in IL-8 during recovery in the oldest groups might be related to another immunological compensative mechanism involved with the rejuvenation of the T cell population in peripheral blood. This hypothesis requires future evaluation with the participation of a larger group of the participants and another molecular analysis of the immune pathways involved in post-effort as well as longer term immune responses.

## 5. Conclusions

In conclusion, it is worth noting that the mechanisms related to the involvement of Th1 and Th2 cell subsets appears to be related to participants’ age. An increase in Th2 cell-related cytokines was found, without similar changes in the cell distribution. These findings are related to the pleiotropic role of the studied cytokines and need further evaluation on the gene level to determine whether protein expression is related to an increase in protein production or just a change in extracellular secretion of these molecules. An interesting finding is the involvement of Th17 cells in the post-effort immune response and its probable role in differentiation into Treg cells. These play an important role as anti-inflammatory regulators of the long-term biological effect of progressive effort, at least in case of young men. The observed negative correlation between the change of selected T cell subsets and participants’ age only at the post-test time point suggests that the older the participant, the lower the biological effect of the effort. Further, it may suggest the better biological adaptation of participants to the physical effort.

In summary, our study demonstrated differential involvement of T cell subsets and suggests that the mechanism of their activation may be related to participants’ age. However, the results are based on limited age groups and further studies are needed to investigate differential T cell activation over wider age groups as well as a much wider age range. An additional study including an analysis of the secretion of cytokines directly from cells isolated from post-effort blood samples is also needed to fully explain the observed relations. The inclusion of a control group of participants who do not perform the endurance test and the addition of a group of women would significantly enrich the study. Another limitation of the study is the lack of Fluorescence Minus One control in phenotyping Th1, Th2, Th17, and Treg cells. However, the assays used in the study provide a cocktail/mix of all antibodies used in the assay in one vial, preventing such a control.

## Figures and Tables

**Table 1:** Theoretical limit of detection for cytokines analyzed in the study.

Cytokine	Theoretical Limit of Detection (pg/mL)
IL-2	2.6
IL-4	4.9
IL-6	2.4
IL-8	3.6
IL-10	3.3
IL-12p70	1.9
IL-17A	18.9
TNF-α	3.7
IFN-γ	3.7

**Table 2:** Baseline characteristics of the participants.

Variable	16 Years Old Group (n = 16)	17 Years Old Group (n = 16)	18 Years Old Group (n = 16)	19 Years Old Group (n = 16)	20 Years Old Group (n = 16)	p_KW_^1^
Height (cm)	180 (178–182)	180 (173–184)	181 (177–184)	178 (172–181)	184 (160–188)	0.820
Weight (kg)	71.0 (64.7–75.5)	69.6 (64.2–76.1)	69.8 (67.0–70.0)	69.2 (63.3–72.3)	75.4 (56.4–81.5)	0.839
FAT (%)	9.9 (7.3–12.2)	7.7 (5.5–11.0)	10.6 (5.3–10.6)	8.2 (7.3–9.8)	9.9 (7.3–12.1)	0.226
FAT MASS (kg)	7.2 (4.8–8.9)	5.7 (3.6–8.0)	7.4 (3.7–7.7)	6.0 (4.8–6.8)	7.9 (4.1–9.8)	0.338
FFM (kg)	63.4 (60.3–67.1)	64.7 (59.8–68.0)	62.4 (59.8–66.3)	63.2 (58.6–65.7)	67.5 (52.3–71.6)	0.835
TBW (kg)	46.4 (44.1–49.1)	47.4 (43.8–49.8)	45.7 (43.8–48.5)	46.3 (42.9–48.1)	49.4 (38.3–52.4)	0.831

^1^ Differences between analyzed age groups were assessed using the Kruskal–Wallis analysis of ranks and the median test (p_KW_—Kruskal–Wallis *p* values). n—number of participants, FAT—percentage of fat, FFM—fat-free mass, TBW—total body water.

**Table 3:** Cardiorespiratory fitness measures of participants during the progressive test until exhaustion.

Variable	16 Years Old Group (n = 16)	17 Years Old Group (n = 16)	18 Years Old Group (n = 16)	19 Years Old Group (n = 16)	20 Years Old Group (n = 16)	p_KW_
VO_2_max (mL/kg/min)	61.0 (58.7–64.7)	60.4 (58.–61.59)	61.9 (57.8–64.6)	60.7 (56.9–63.2)	60.2 (57.9–61.1)	0.721
HR_max_ (beats/min)	198 (188–201)	197 (191–203)	192 (189–201)	192 (188–200)	196 (186–200)	0.762
AT (beats/min)	160 (154–169)	164 (161–170)	166 (159–174)	160 (154–168)	162 (153–169)	0.522
RQ	1.06 (1.05–1.08)	1.06 (1.05–1.08)	1.07 (1.05–1.08)	1.04 (0.95–1.06)	1.07 (1.05–1.10)	0.050
RC	172 (165–177)	175 (172–187)	175 (169–186)	175 (169–183)	172 (168–180)	0.735
V_E_ (L/min)	146.7 (129.5–156.4)	155.7 (139.2–175.1)	150.9 (141.0–165.1)	149.1 (139.8–155.6)	152.9 (134.7–162.6)	0.565
MVV (L/min)	190.4 (182.4–197.7)	189.5 (182.9–202.7)	188.2 (178.6–194.6)	187.2 (177.7–195.5)	192.0 (183.2–204.3)	0.824
MET (mL/kg/min)	17.3 (16.6–18.4)	17.8 (17.1–18.8)	17.7 (17.1–18.9)	17.2 (16.7–18.6)	17.3 (16.7–17.9)	0.433
Rf	58.2 (56.8–65.7)	66.4 (57.6–68.0)	60.7 (53.4–68.5)	59.1 (56.1–65.0)	56.4 (54.3–60.6)	0.320

The analyses were performed using a state-of-the-art breath by breath gas exchange data analyzer Quark CPET (Cosmed, Albano Laziale, Italy). Differences between analyzed age groups were assessed using the Kruskal-Wallis analysis of ranks and the median test (pKW—Kruskal-Wallis *p* values). n—number of participants, VO_2_max—maximum oxygen uptake; HRmax—maximum heart rate; AT—anaerobic threshold; RQ—respiratory quotient (volume ratio of emitted CO_2_ to oxygen uptake); RC—respiratory compensation; VE—minute ventilation; MVV—maximal voluntary ventilation; MET—metabolic equivalent; Rf—respiratory frequency.

**Table 4:** White blood cell (WBC), and lymphocyte (LYM) counts of studied participants’ blood samples.

Variable	Time Point	16 Years Old Group (n = 16)	17 Years Old Group (n = 16)	18 Years Old Group (n = 16)	19 Years Old Group (n = 16)	20 Years Old Group (n = 16)
WBC (10^9^/L)	p_F_^1^	<0.001	<0.001	<0.001	<0.001	<0.001
	pre-test	5.8 ^aa^ (5.2–6.6)	5.2 ^aaa^ (4.5–5.8)	5.2 ^aa^ (4.6–6.3)	5.4 ^aaa^ (4.8–5.7)	5.3 ^aaa^ (4.7–6.3)
	post-test	8.7 ^bbb^ (7.4–10.3)	8.4 ^bbb^ (7.0–9.6)	8.6 ^bbb^ (7.0–9.9)	8.7 ^bbb^ (7.2–11.4)	10.9 ^bbb^ (8.9–12.3)
	recovery	5.7 (5.2–6.0)	5.7 (4.6–6.3)	5.0 (4.4–6.0)	5.6 (5.2–6.0)	5.6 (4.6–6.6)
LYM (10^9^/L)	p_F_	0.005	<0.001	<0.001	<0.001	<0.001
	pre-test	2.4 (2.1–3.0)	2.0 ^aaa^ (1.7–2.2)	1.9 (1.8–2.2)	2.2 ^aaa^ (2.0–2.2)	2.1 ^aaa^ (1.7–2.2)
	post-test	3.8 ^bb^ (2.7–4.4)	4.0 ^bbb^ (2.8–4.9)	3.7 ^bbb^ (2.6–4.5)	4.0 ^bbb^ (2.8–4.5)	4.2 ^bbb^ (3.9–4.7)
	recovery	2.3 (1.9–2.7)	1.8 (1.6–2.2)	1.8 (1.6–2.0)	2.1 (1.9–2.4)	1.9 (1.6–2.5)

^1^ Significance levels of differences observed between analyzed time points (pre-test vs. post-test vs. recovery) were assessed using Friedman’s analysis of variance for repeated measures (pF—Friedman’s ANOVA *p* values) followed by post hoc Dunn’s test with Bonferroni correction. Post hoc *p* values: ^aa^ *p* < 0.01, ^aaa^ *p* < 0.001 for pre-test vs. post-test; ^bb^ *p* < 0.01, ^bbb^ *p* < 0.001 for post-test vs. recovery.

**Table 5:** Coefficients of correlation between analyzed variables and the age of the participants.

Variables Correlated	Pre-Test		Post-Test		Recovery	
	R	*p*	R	*p*	R	*p*
Th1 cells (%) & age	0.11	0.347	0.10	0.362	0.06	0.570
Th2 cells (%) & age	−0.17	0.143	−0.28	0.012	−0.08	0.502
Th17 cells (%) & age	0.04	0.753	0.11	0.312	−0.05	0.668
Treg cells (%) & age	−0.01	0.933	−0.24	0.030	0.04	0.711
IL-2 (pg/mL) & age	−0.20	0.081	0.18	0.101	−0.23	0.041
*IL-4 (pg/mL) & age*	*0.04*	*0.758*	−*0.07*	*0.519*	−*0.09*	*0.426*
IL-6 (pg/mL) & age	−0.04	0.751	−0.23	0.036	−0.24	0.035
IL-8 (pg/mL) & age	−0.08	0.489	−0.15	0.188	−0.32	0.003
*IL-10 (pg/mL) & age*	*0.17*	*0.141*	*0.28*	*0.011*	*0.42*	*<0.001*
*IL-12p70 (pg/mL) & age*	−*0.36*	*0.001*	−*0.10*	*0.360*	0.23	0.040
*IL-17A (pg/mL) & age*	*0.00*	*1.000*	*0.29*	*0.010*	−*0.28*	*0.012*
*TNF (pg/mL) & age*	−*0.02*	*0.871*	−*0.38*	*0.001*	*0.30*	*0.007*
IFN (pg/mL) & age	0.02	0.889	0.16	0.155	0.02	0.848
WBC (10^9^/L) & age	−0.07	0.512	0.22	0.052	−0.03	0.759
LYM (10^9^/L) & age	−0.23	0.037	0.13	0.259	−0.05	0.675

The analysis of correlations between concentrations of every analyzed variable with participants’ age was performed using the Spearman rank correlation coefficient test. The values in italic are not confident as cytokines are not within the range of the assay.

## Supplementary Materials

The following are available online at https://www.mdpi.com/2077-0383/9/6/1795/s1, Figure S1: Median level of (a) interleukin-4 (IL-4), (b) interleukin-10 (IL-10), (c) interleukin-12p70 (IL-12p70), (d) interleukin-17A (IL-17A) and (e) tumor necrosis factor alpha (TNF-α) of participants’ plasma samples, Table S1: Raw data obtained for each individual participant during the study, Table S2: Median level of percentage of lymphocytes, T lymphocytes, Th lymphocytes, Tc lymphocytes, NK cells and B lymphocytes of participants’ blood samples, Table S3: Linear regression parameters for age effects.
