# Supplementary material for: Differential Th Cell-Related Immune Responses in Young Physically Active Men after an Endurance Effort
Source: J Clin Med. 2020 Jun 9;9(6):1795. doi: 10.3390/jcm9061795 (PMC7356896; doi:10.3390/jcm9061795)
Supplement: Supplementary file 1 [file jcm-09-01795-s001.zip › supple/jcm-779803supple.pdf]

a)

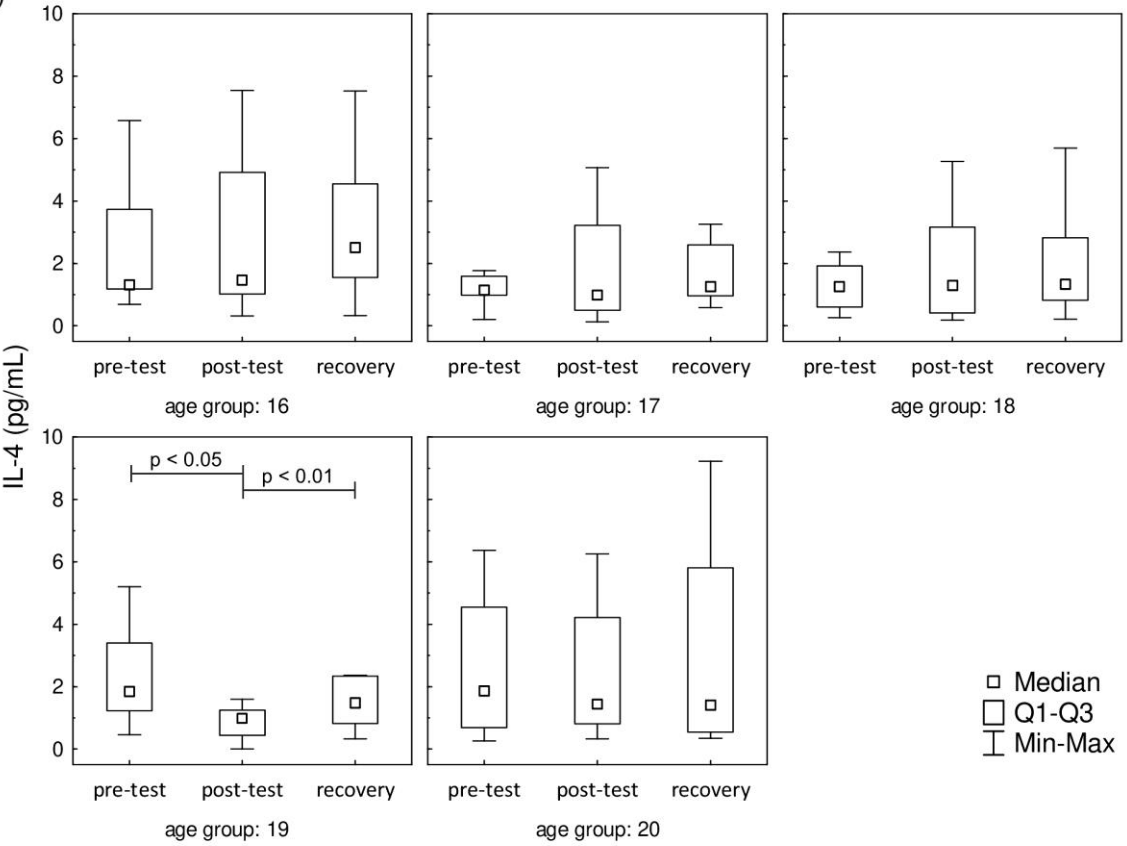
IL-4 (pg/mL)
0
2
4
6
8
10
pre-test
post-test
recovery
age group: 16
age group: 17
age group: 18
age group: 19
age group: 20
p < 0.05
p < 0.01
Median
Q1-Q3
Min-Max

b)

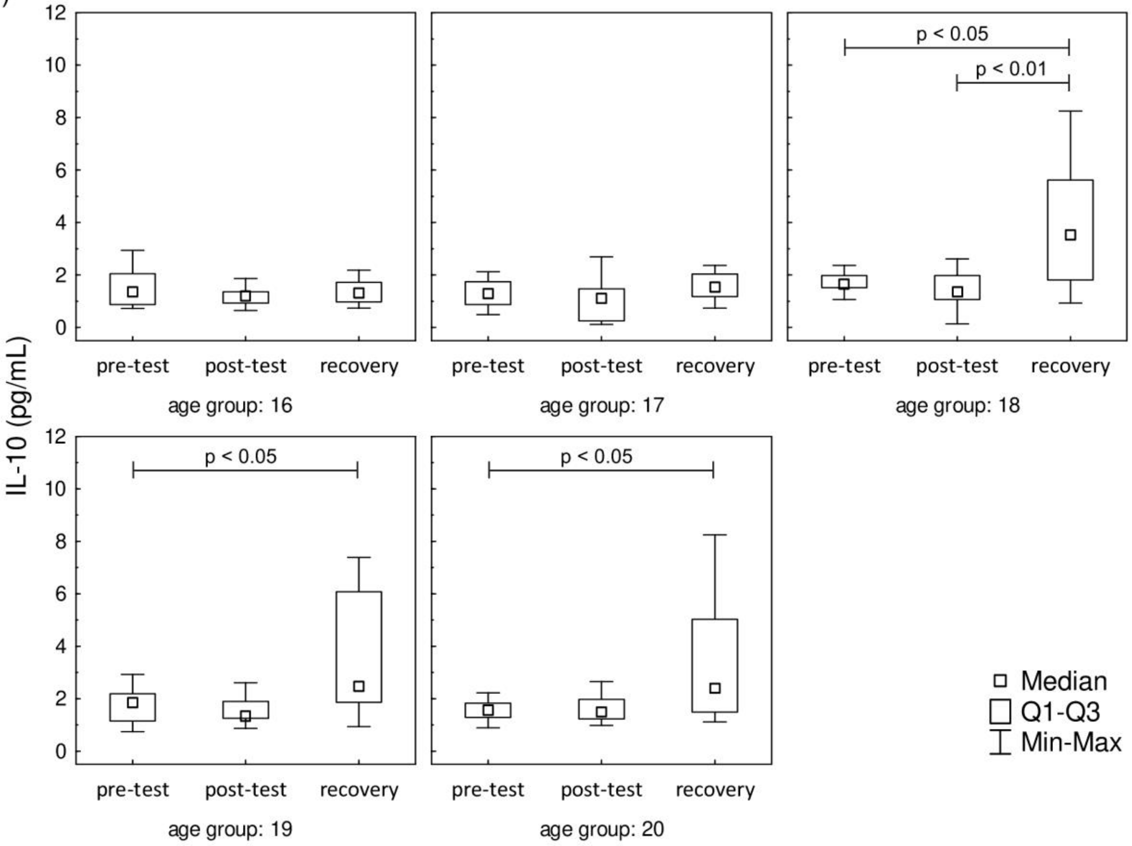
IL-10 (pg/mL)
0
2
4
6
8
10
12
pre-test
post-test
recovery
age group: 16
age group: 17
age group: 18
age group: 19
age group: 20
p < 0.05
p < 0.01
p < 0.05
p < 0.05
Median
Q1-Q3
Min-Max

c)

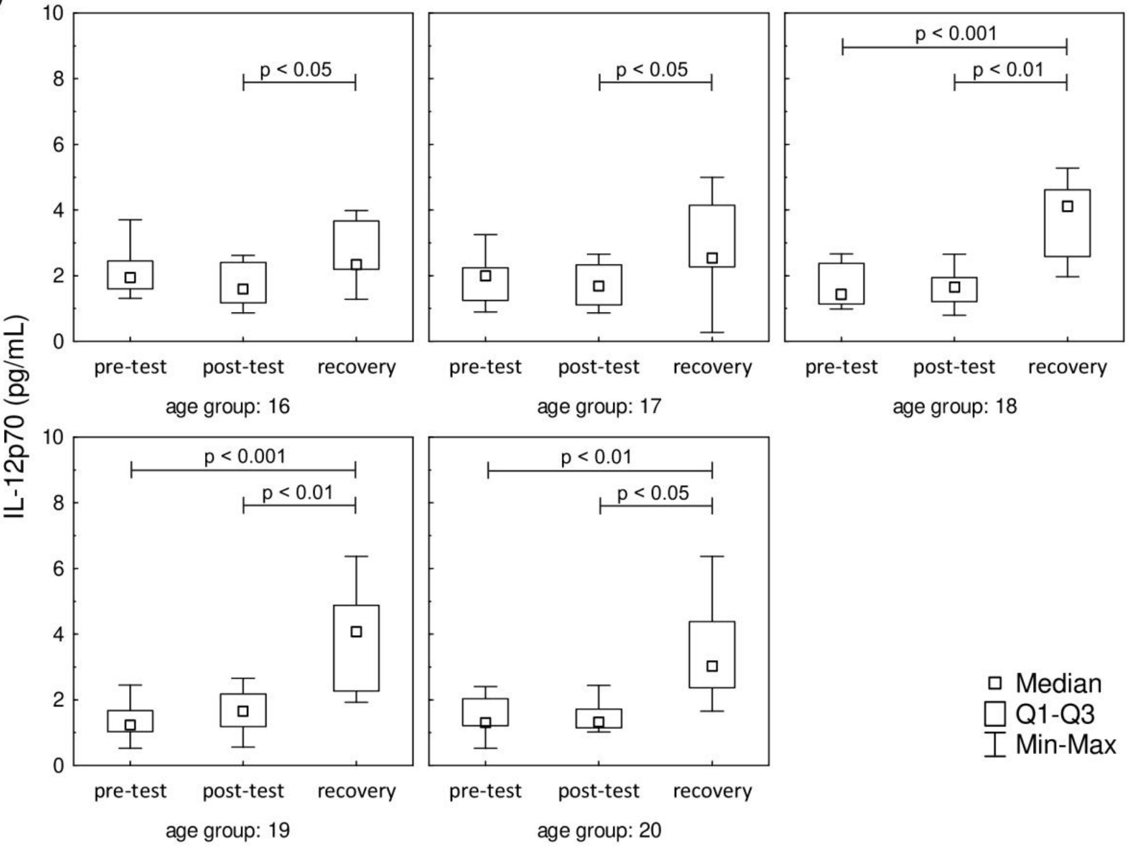
IL-12p70 (pg/mL)
p < 0.05
p < 0.05
p < 0.001
p < 0.01
p < 0.001
p < 0.01
p < 0.01
p < 0.05
pre-test
post-test
recovery
age group: 16
age group: 17
age group: 18
age group: 19
age group: 20
Median
Q1-Q3
Min-Max

d)

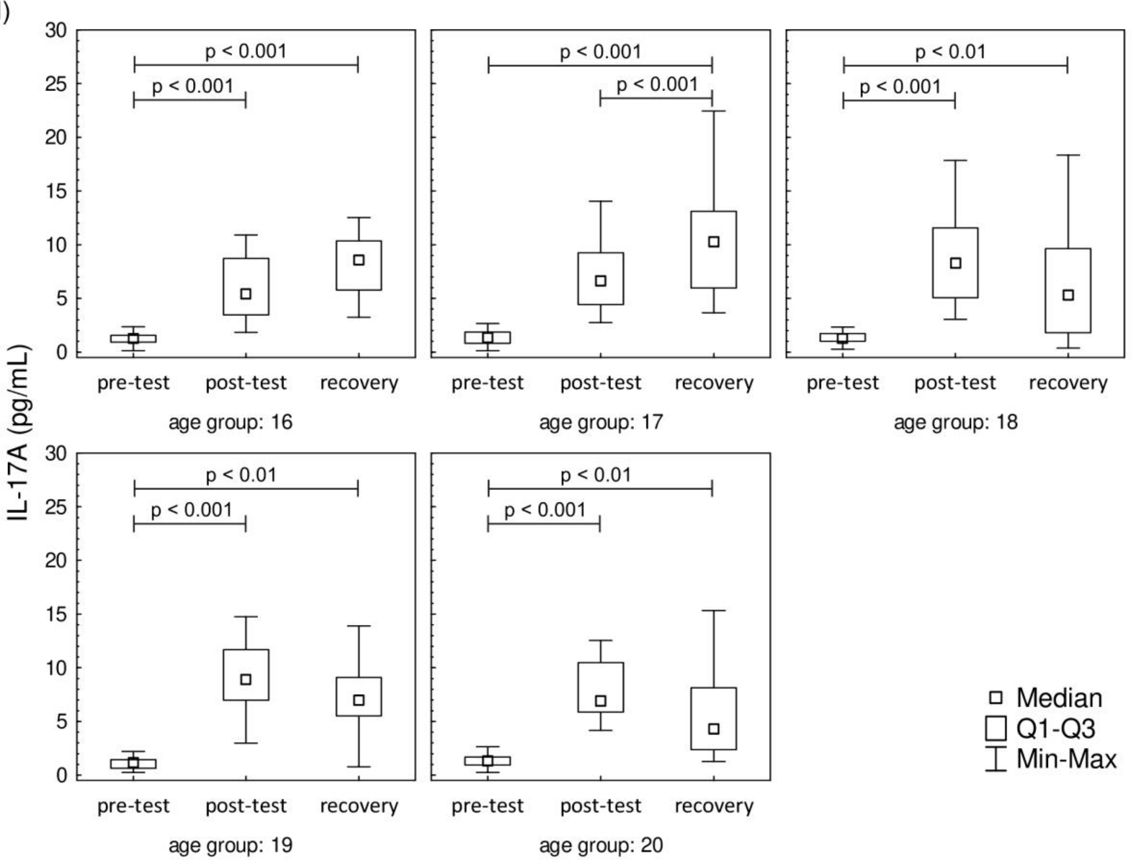
IL-17A (pg/mL)
p < 0.001
p < 0.001
p < 0.001
p < 0.001
p < 0.01
p < 0.001
p < 0.01
p < 0.001
p < 0.01
p < 0.001
pre-test
post-test
recovery
age group: 16
age group: 17
age group: 18
age group: 19
age group: 20
Median
Q1-Q3
Min-Max

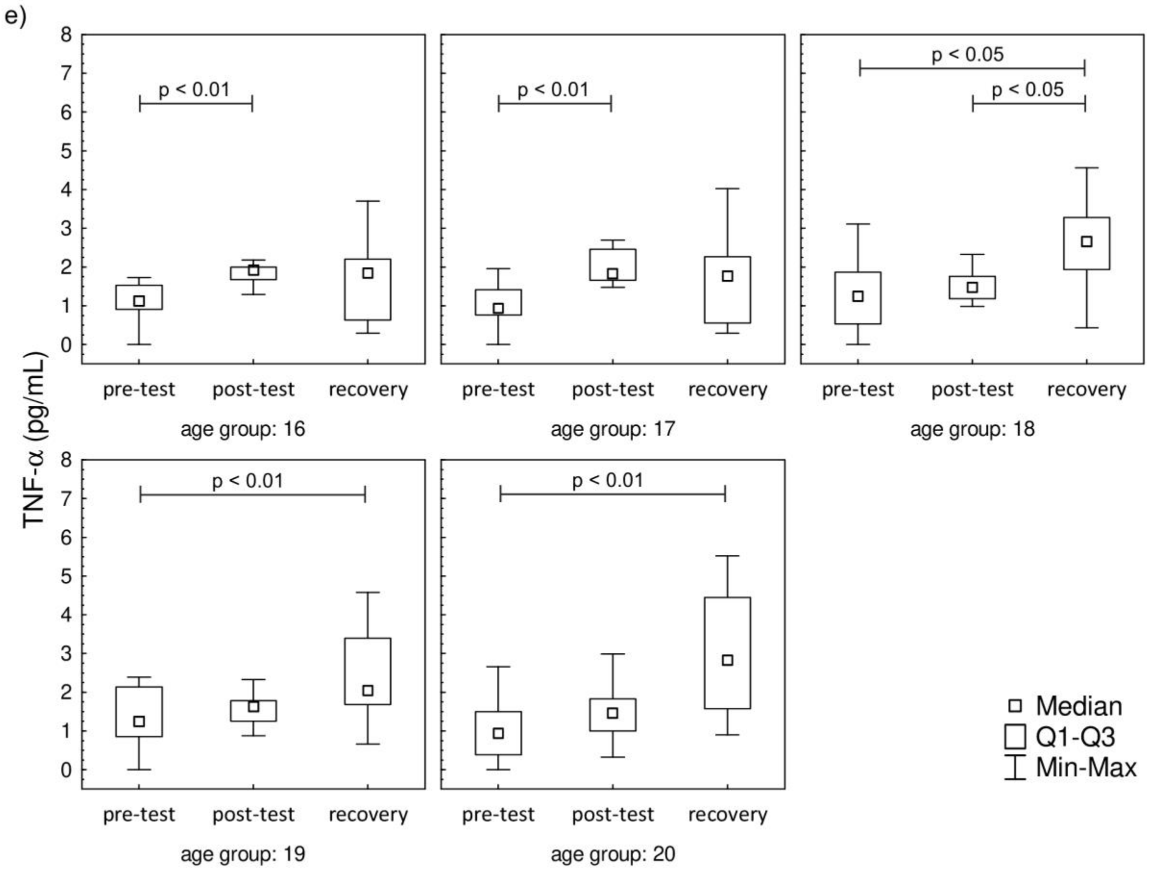

Figure 1. Median level of (**a**) interleukin-4 (IL-4), (**b**) interleukin-10 (IL-10), (**c**) interleukin-12p70 (IL-12p70), (**d**) interleukin-17A (IL-17A) and (**e**) tumor necrosis factor alpha (TNF-α) of participants' plasma samples. Significance levels of differences observed between analyzed time points (pre-test vs. post-test vs. recovery) were assessed using Friedman's analysis of variance for repeated measures followed by post-hoc Dunn's test with Bonferroni correction.

Table 2. Median level of percentage of lymphocytes, T lymphocytes, Th lymphocytes, Tc lymphocytes, NK cells and B lymphocytes of participants' blood samples.

**Variable**	**Time point**	**16 years old group (n = 16)**	**17 years old group (n = 16)**	**18 years old group (n = 16)**	**19 years old group (n = 16)**	**20 years old group (n = 16)**
Lymphocytes (%)	p_F [1]	0.005	< 0.001	0.003	0.050	0.003
	pre-test	24.6^{aa} (19.5–28.3)	20.0^{aa} (18.7–25.8)	16.9^{a} (13.6–19.3)	21.3 (18.5–23.1)	25.6 (24.1–29.2)
	post-test	27.6 (25.8–31.4)	26.3^{bbb} (22.9–31.2)	19.7 (17.7–23.3)	23.2 (19.1–25.6)	29.1^{bb} (26.1–31.9)
	recovery	26.7 (21.7–29.0)	17.5 (15.9–18.6)	20.8^{cc} (19.2–24.5)	21.1 (18.8–23.4)	21.6 (16.5–28.6)
T lymphocytes (%)	p_F	< 0.001	< 0.001	< 0.001	< 0.001	< 0.001
	pre-test	66.7^{aa} (60.3–73.8)	68.3^{a} (64.4–72.9)	69.2 (62.9–73.2)	64.6 (58.6–71.4)	70.9 (62.4–72.4)
	post-test	58.2^{bbb} (51.2–65.3)	59.4^{bbb} (53.0–65.9)	62.8^{bbb} (55.5–67.6)	59.7^{bbb} (51.4–67.6)	63.3^{bbb} (58.6–71.3)
	recovery	71.1 (63.9–76.9)	71.3 (66.8–74.1)	72.0^{cc} (66.8–77.9)	71.4^{c} (66.0–77.5)	72.1 (66.4–76.8)
	p_F	< 0.001	< 0.001	< 0.001	< 0.001	< 0.001

Th lymphocytes (%)	pre-test	54.5[aa] (45.4–61.4)	54.7[aa] (50.5–60.0)	52.5[aa] (43.0–61.8)	53.2[aa] (45.8–62.4)	55.3[a] (46.3–59.3)
	post-test	45.8[bbb] (43.2–53.7)	50.7[bbb] (43.9–56.7)	45.4[bbb] (36.6–53.9)	45.9[bbb] (38.1–58.7)	47.0[bbb] (42.1–53.4)
	recovery	56.3 (51.9–61.5)	56.3 (49.6–64.4)	55.4 (44.2–62.2)	59.0 (50.0–65.6)	57.7 (51.2–60.9)
	p_F	< 0.001	0.003	0.002	0.002	< 0.001
Tc lymphocytes (%)	pre-test	33.9 (27.8–43.6)	34.0 (29.4–41.0)	36.2 (32.2–42.6)	34.1 (29.7–44.6)	35.6[a] (32.3–41.4)
	post-test	39.4[bbb] (32.9–48.2)	35.5[bb] (32.9–42.0)	40.6[bb] (36.0–44.5)	37.7[bb] (31.6–46.9)	38.5[bbb] (36.9–44.2)
	recovery	33.6 (28.2–41.4)	32.1 (26.6–40.5)	35.6 (31.3–41.2)	33.6 (27.2–38.1)	34.6 (31.4–37.8)
	p_F	< 0.001	< 0.001	< 0.001	< 0.001	< 0.001
NK cells (%)	pre-test	18.8[aa] (12.6–25.1)	18.2[aa] (16.0–22.7)	16.8[a] (13.9–21.3)	18.8[aa] (16.5–25.2)	18.0 (13.6–24.6)
	post-test	30.9[bbb] (20.1–35.8)	27.6[bbb] (21.2–33.8)	27.1[bbb] (19.5–31.6)	27.2[bbb] (19.9–32.7)	24.3[bbb] (16.9–26.9)
	recovery	14.6[c] (8.5–20.5)	11.3 (10.2–16.4)	13.1[c] (10.5–15.6)	13.0 (9.7–19.8)	11.1[c] (8.3–18.2)
	p_F	0.002	0.019	< 0.001	0.018	0.003
B lymphocytes (%)	pre-test	11.4[a] (8.6–14.2)	10.2 (7.7–12.2)	11.0[aa] (9.2–12.7)	9.9 (9.0–11.9)	10.6 (9.4–13.2)
	post-test	9.5[bb] (7.4–12.9)	8.7[b] (7.2–10.7)	8.7[bbb] (7.2–10.8)	8.8[b] (7.9–10.9)	9.2[bb] (7.4–10.5)
	recovery	11.7 (10.0–14.3)	11.6 (8.7–14.0)	11.6 (9.9–14.3)	11.3 (10.2–14.3)	12.9 (12.0–14.1)

[1] Significance levels of differences observed between analyzed time points (pre-test vs. post-test vs. recovery) were assessed using Friedman's analysis of variance for repeated measures (pF - Friedman's ANOVA p values) followed by post-hoc Dunn's test with Bonferroni correction. Post-hoc p values: [a] $p < 0.05$, [aa] $p < 0.01$ for pre-test vs. post-test; [b] $p < 0.05$, [bb] $p < 0.01$, [bbb] $p < 0.001$ for post-test vs. recovery; [c] $p < 0.05$, [cc] $p < 0.01$ for pre-test vs. recovery. n – number of participants. Whole blood lymphocyte subsets were determined using a BD Multitest™ IMK kit (BD Biosciences, San Jose, CA, USA). The expression of surface markers was determined according to the manufacturer's protocol. Briefly, an antibody cocktail was used to determine the percentage of T-lymphocyte subsets in erythrocyte-lysed blood samples. The cocktail contained antibodies including fluorescein isothiocyanate (FITC)-labelled anti-CD3, clone SK7; phycoerythrin (PE)-labelled anti-CD8, clone SK1; peridinin chlorophyll protein (PerCP)-labelled anti-CD45, clone 2D1 (HLe-1) and allophycocyanin (APC)-labelled anti-CD4. For each sample, the fluorescence signal of at least 10^4 total events were measured.

Table 3. Linear regression parameters for age effects.

Variable analyzed	Time point	Linear regression equation	R value	R^2 value	p value
Th1 cells (%)	pre-test	$Th1 = 0.408 \times age + 10.532$	0.135	0.018	0.232
	post-test	$Th1 = 0.456 \times age + 16.118$	0.138	0.019	0.221
	recovery	$Th1 = 0.575 \times age + 15.488$	0.161	0.026	0.154
Th2 cells (%)	pre-test	$Th2 = -0.694 \times age + 24.378$	-0.257	0.066	0.021
	post-test	$Th2 = -0.702 \times age + 25.899$	-0.325	0.106	0.003
	recovery	$Th2 = -0.228 \times age + 18.169$	-0.101	0.010	0.371
Th17 cells (%)	pre-test	$Th17 = 0.221 \times age + 14.267$	0.056	0.003	0.624
	post-test	$Th17 = 0.368 \times age + 18.205$	0.144	0.021	0.202
	recovery	$Th17 = -0.300 \times age + 31.988$	0.069	0.005	0.543
Treg cells (%)	pre-test	$Treg = -0.022 \times age + 4.995$	0.019	< 0.001	0.864
	post-test	$Treg = -0.219 \times age + 8.696$	0.160	0.026	0.155
	recovery	$Treg = 0.1332 \times age + 8.838$	0.043	0.002	0.707
IL-2 (pg/mL)	pre-test	$IL\text{-}2 = -0.237 \times age + 7.155$	0.170	0.029	0.132
	post-test	$IL\text{-}2 = 0.694 \times age - 2.983$	0.230	0.053	0.040
	recovery	$IL\text{-}2 = -0.702 \times age + 21.526$	0.229	0.052	0.041
IL-4 (pg/mL)	pre-test	$IL\text{-}4 = 0.081 \times age + 0.591$	0.068	0.005	0.548
	post-test	$IL\text{-}4 = -0.214 \times age + 5.953$	0.140	0.020	0.216
	recovery	$IL\text{-}4 = -0.020 \times age + 2.913$	0.012	< 0.001	0.917
IL-6 (pg/mL)	pre-test	$IL\text{-}6 = 0.034 \times age + 1.164$	0.056	0.003	0.622
	post-test	$IL\text{-}6 = -0.482 \times age + 12.498$	0.261	0.068	0.019
	recovery	$IL\text{-}6 = -1.198 \times age + 31.320$	0.265	0.070	0.017
IL-8 (pg/mL)	pre-test	$IL\text{-}8 = -0.061 \times age + 5.112$	0.043	0.002	0.706
	post-test	$IL\text{-}8 = -0.500 \times age + 17.979$	0.157	0.025	0.164
	recovery	$IL\text{-}8 = -1.348 \times age + 34.122$	0.372	0.138	< 0,001
IL-10 (pg/mL)	pre-test	$IL\text{-}10 = 0.060 \times age + 0.487$	0.153	0.023	0.174
	post-test	$IL\text{-}10 = 0.116 \times age - 0.678$	0.251	0.063	0.025
	recovery	$IL\text{-}10 = 0.603 \times age - 8.043$	0.413	0.170	< 0.001
IL-12p70 (pg/mL)	pre-test	$IL\text{-}12p70 = -0.135 \times age + 4.175$	0.281	0.079	0.011
	post-test	$IL\text{-}12p70 = -0.045 \times age + 2.482$	0.109	0.012	0.334
	recovery	$IL\text{-}12p70 = 0.230 \times age - 0.816$	0.251	0.063	0.025
IL-17A (pg/mL)	pre-test	$IL\text{-}17A = 0.001 \times age + 1.231$	0.003	< 0.001	0.975
	post-test	$IL\text{-}17A = 0.574 \times age - 2.532$	0.227	0.051	0.043
	recovery	$IL\text{-}17A = -0.765 \times age + 21.319$	0.252	0.063	0.024
TNF-α (pg/mL)	pre-test	$TNF\text{-}\alpha = -0.010 \times age + 1.370$	0.018	< 0.001	0.975
	post-test	$TNF\text{-}\alpha = -0.124 \times age + 3.940$	0.313	0.098	0.005
	recovery	$TNF\text{-}\alpha = 0.319 \times age - 3.394$	0.306	0.094	0.006
INF-γ (pg/mL)	pre-test	$IFN\text{-}\gamma = -0.200 \times age + 2.730$	0.027	< 0.001	0.810
	post-test	$IFN\text{-}\gamma = 0.516 \times age + 0.824$	0.150	0.022	0.185
	recovery	$IFN\text{-}\gamma = 0.165 \times age + 9.900$	0.060	0.003	0.598

The regression analysis was performed using STATISTICA (data analysis software system), version 13 software (2017; TIBCO Software Inc., Palo Alto, CA, USA; http://statistica.io).
